# *Klebsiella oxytoca* expands in cancer cachexia and acts as a gut pathobiont contributing to intestinal dysfunction

**DOI:** 10.1038/s41598-018-30569-5

**Published:** 2018-08-17

**Authors:** Sarah A. Pötgens, Hélène Brossel, Martina Sboarina, Emilie Catry, Patrice D. Cani, Audrey M. Neyrinck, Nathalie M. Delzenne, Laure B. Bindels

**Affiliations:** 1 Metabolism and Nutrition Research Group, Louvain Drug Research Institute, Université catholique de Louvain, Brussels, Belgium; 2 Walloon Excellence in Life Sciences and BIOtechnology (WELBIO), Louvain Drug Research Institute, Université catholique de Louvain, Brussels, Belgium

## Abstract

Cancer cachexia is a complex multi-organ syndrome characterized by body weight loss, weakness, muscle atrophy and fat depletion. With a prevalence of 1 million people in Europe and only limited therapeutic options, there is a high medical need for new approaches to treat cachexia. Our latest results highlighted microbial dysbiosis, characterized by a bloom in *Enterobacteriaceae* and altered gut barrier function in preclinical models of cancer cachexia. They also demonstrated the potential of targeting the gut microbial dysbiosis in this pathology. However, the exact mechanisms underlying the gut microbiota-host crosstalk in cancer cachexia remain elusive. In this set of studies, we identified *Klebsiella oxytoca* as one of the main *Enterobacteriaceae* species increased in cancer cachexia and we demonstrated that this bacteria acts as a gut pathobiont by altering gut barrier function in cachectic mice. Moreover, we propose a conceptual framework for the lower colonization resistance to *K*. *oxytoca* in cancer cachexia that involves altered host gut epithelial metabolism and host-derived nitrate boosting the growth of the gut pathobiont. This set of studies constitutes a strong progression in the field of gut microbiota in cancer cachexia, by dissecting the mechanism of emergence of one bacterium, *K*. *oxytoca*, and establishing its role as a gut pathobiont in this severe disease.

## Introduction

Cancer cachexia is a complex multi-organ syndrome characterized by body weight loss, weakness, muscle atrophy and fat depletion^1–3^. These metabolic alterations are thought to be driven by pro-inflammatory mediators arising from the tumor-immune system crosstalk as well as by tumor-derived catabolic factors^1,4^. Clinically, cachexia can affect up to 70% of cancer patients, depending on the cancer type^1^. Cachexia leads to an increase in morbidity and mortality rates and a reduction in anti-cancer treatment tolerance^5,6^. Currently, limited therapeutic options exist for this serious medical challenge and new approaches to tackle this syndrome, including innovative and scientifically relevant nutritional tools, are needed^3,7,8^. In this context, targeting the gut microbiota represents an exciting opportunity for this public health issue^9,10^.

Relationships between gut microbiota and cancer have been investigated for years^11,12^. Our research over the last ten years has evidenced the existence of a crosstalk between the gut, the microbes it harbors and metabolic alterations occurring during cancer. For instance, we showed in 2012 that restoring the lactobacilli levels through the administration of lactobacilli counteracted muscle atrophy and decreased systemic inflammation in a mouse model of leukemia and cachexia^13^. This decrease in muscle atrophy upon lactobacilli administration was confirmed in 2016 in cachectic mice with colon cancer^14^. We also reported several times that nutritional interventions targeting the microbiota, such as prebiotics or probiotics, decreased cancer progression, reduced morbidity and fat mass loss, and/or increased survival of cachectic mice with leukemia^15–17^. Importantly, we highlighted a common microbial signature (characterized mainly by an increase in *Enterobacteriaceae*) in preclinical models of cancer cachexia^15,16^. Alongside these changes, we found deep perturbations of the gut barrier functions and intestinal morphology in cachectic mice. The gut barrier dysfunctions consisted in increased gut permeability and decreased cell renewal and mucosal immunity^15,18^. Such alterations were strongly correlated with the cachectic features, occurred independently of anorexia and were alleviated using antibodies targeting interleukin 6. Gut dysfunction was not alleviated neither by treatments with an anti-inflammatory bacterium (*Faecalibacterium prausnitzii*) nor with gut peptides involved in intestinal cell renewal (i.e. teduglutide, a glucagon-like peptide-2 analogue)^18^.

Altogether, our studies reveal a previously unexpected link between cancer, cachexia and the gut microbiota. However, several questions remain open. Which *Enterobacteriaceae* are increased? Which mechanisms explain this increase? Does this bloom of *Enterobacteriaceae* contribute to the intestinal alterations associated with cachexia? To answer these questions, we isolated *Enterobacteriaceae* members from cachectic mice and we evaluated whether the selected isolated bacteria promote gut dysfunctions *in vivo*. We also investigated the potential mechanisms of expansion of *Enterobacteriaceae* in cachectic mice.

## Results

### *Klebsiella oxytoca* is increased in tumor-bearing mice with cachexia independently of anorexia

**Figure 1:**
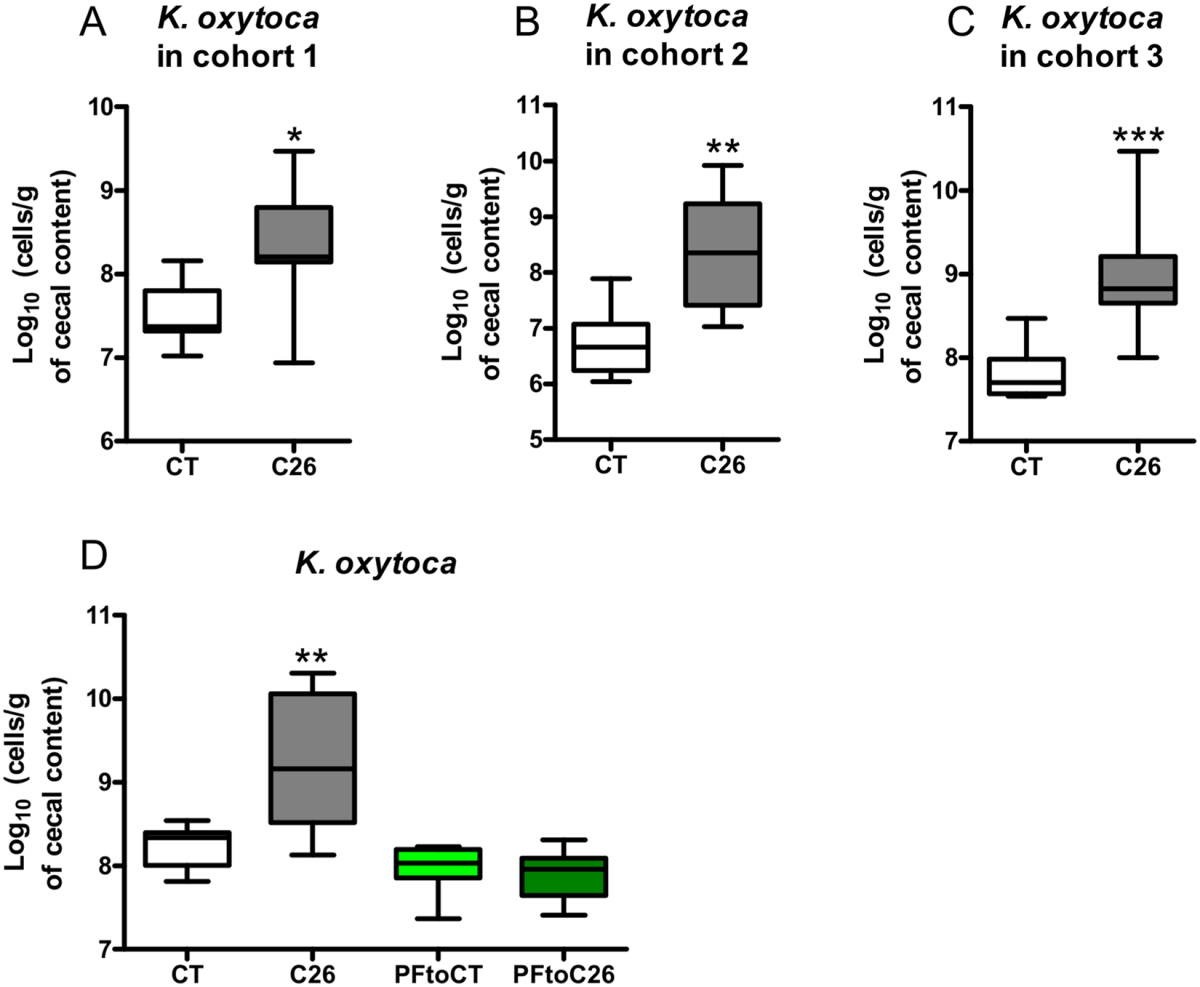
*Klebsiella oxytoca* is increased in tumor-bearing mice with cachexia independently of anorexia. (**A**–**C**) Cecal *K*. *oxytoca* levels in three independent cohorts of sham-injected mice (CT) and C26-injected mice (C26), n = 7–8 for each group of mice. (**D**) Cecal *K*. *oxytoca* levels in sham-injected mice (CT), C26-injected mice (C26), healthy mice pair-fed to sham-injected mice (PFtoCT) and healthy mice pair-fed to C26-injected mice (PFtoC26), n = 7–8. Data are presented as whiskers plots with minimal and maximal values. *p < 0.05; **p < 0.01; ***p < 0.001.

The C26 cachexia model consists of a subcutaneous injection of colon carcinoma cells^19^. Mice develop a relatively small tumor mass and display a decreased food intake and loss of body weight, due to both muscle atrophy and adipose tissue loss^18^. Feces from four cachectic mice were cultured on a coliform-selective medium and 15–16 isolates per mouse were identified based on DNA sequencing of their 16S rRNA encoding gene, with a majority of isolates classified as *K*. *oxytoca* (Table 1). This increase in *K*. *oxytoca* upon cancer cachexia was confirmed using qPCR in three independent mouse cohorts (Fig. 1A–C).

**Table 1:** Identification of bacterial isolates recovered from feces of four cachectic mice cultivated on a selective medium for coliform bacteria.

Identification	Prevalence				Average prevalence (%)
*Klebsiella oxytoca*	15/16	9/15	15/16	12/16	84%
*Enterobacter sp*.	1/16	6/15	1/16	4/16	19%

Undernutrition and malnutrition have been shown to be associated with microbial dysbiosis^20^. To assess the role of a reduced caloric intake in the increased *K*. *oxytoca* levels found in cachectic mice, we included pair-fed animals in a similar *in vivo* experiment. Two additional groups of healthy mice were included. One was pair-fed to the CT group (PFtoCT) and the other to the C26 group (PFtoC26). The PFtoCT group serves as a control for the stress related to the pair-feeding procedure. The comparison of the PFtoCT and the PFtoC26 groups allows the strict evaluation of the caloric restriction consequences. As expected, C26 mice displayed increased cecal *K*. *oxytoca* levels compared to CT mice. However, mice pair-fed to cachectic mice (PFtoC26) exhibited levels of *K*. *oxytoca* similar to the ones present in mice pair-fed to the control mice (PFtoCT, Fig. 1D). Hence, we concluded that the reduced food intake alone does not drive the increase in *K*. *oxytoca* observed in cachectic mice.

### Mechanisms of emergence of *Klebsiella oxytoca* in cachectic mice

**Figure 2:**
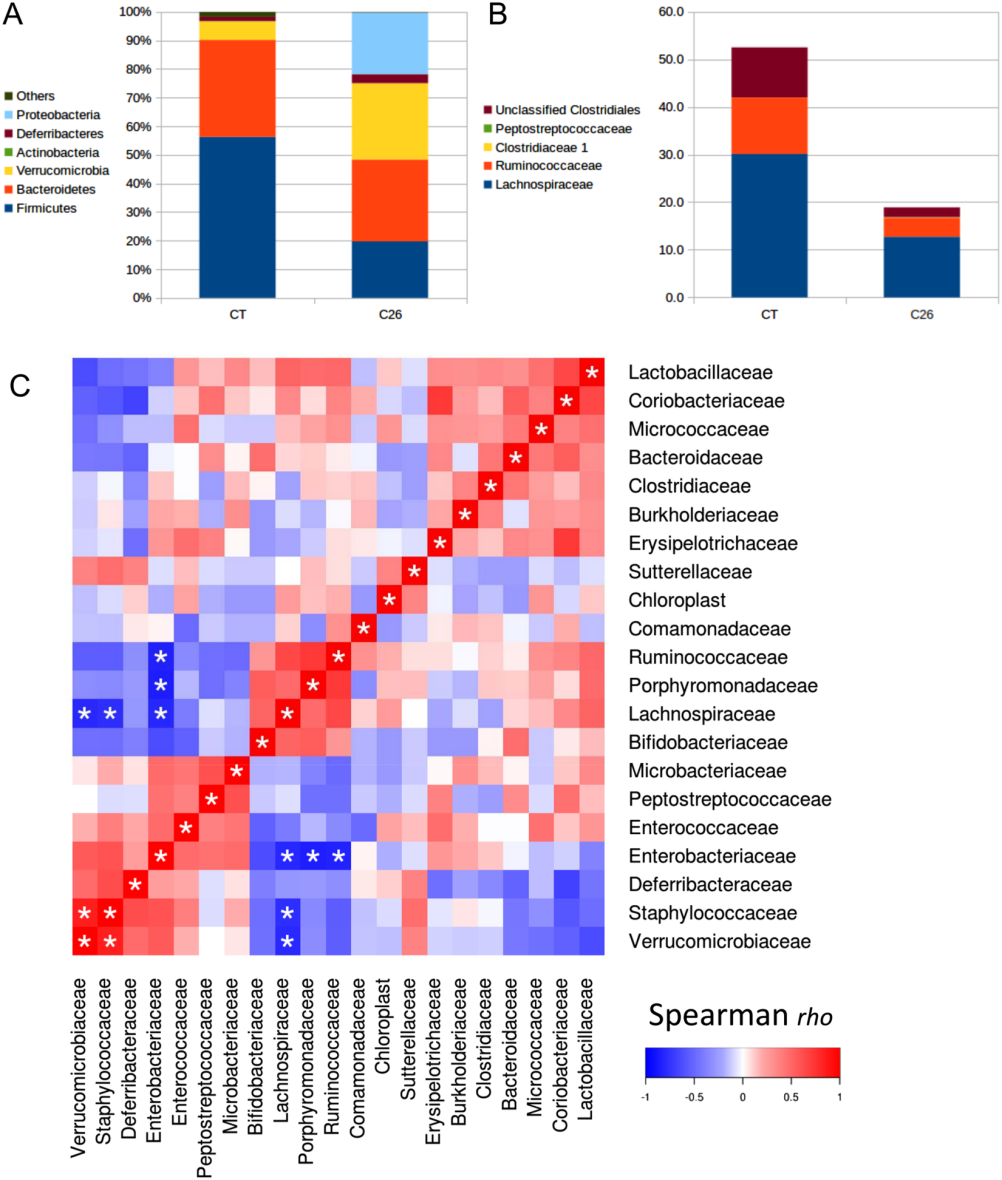
Expansion in Proteobacteria at the expense of the *Ruminococcaceae* and *Lachnospiraceae* families in cachectic mice. (**A**) Stacked plots displaying the mean of the relative abundance of each phylum. (**B**) Stacked plots displaying the mean of the relative abundance of each family within the Clostridiales order. (**C**) Heat map representation of the Spearman rank-order correlation coefficient *rho* between all bacterial families detected by Illumina sequencing of the 16S rRNA gene. *q < 0.05. Mean, SEM, p- and q-values for all phyla and families are presented in Table S1, n = 5 for sham-injected mice (CT) and n = 8 for C26-injected mice (C26).

We have then investigated the mechanisms underlying the expansion of *K*. *oxytoca* in these mice. We know from the 1950s that the gut microbiota can confer “colonization resistance” against members of the family *Enterobacteriaceae* (phylum Proteobacteria)^21^. Therefore, we looked for bacteria which abundance would be reduced in cancer cachexia. At the phylum level, we observed that the expansion of Proteobacteria occurs at the expense of the Firmicutes (Fig. 2A and Table S1). Such drastic decrease in the Firmicutes phylum (from 56% to 20%) was mainly explained by a decrease in the Clostridiales order (from 53% to 19%) and more specifically by a decreased abundance of the *Ruminococcaceae* and *Lachnospiraceae* families (Fig. 2B and Table S1). Of note, these two families, alongside the *Porphyromonadaceae*, are the only ones that were strongly negatively associated with the *Enterobacteriaceae* levels in a multiple correlation analysis (Spearman *rho* of −0.81, −0.84 and −0.85, respectively, all q-value < 0.05, Fig. 2C). Similar information was retrieved from co-abundance network analysis (Fig. S1).

**Figure 3:**
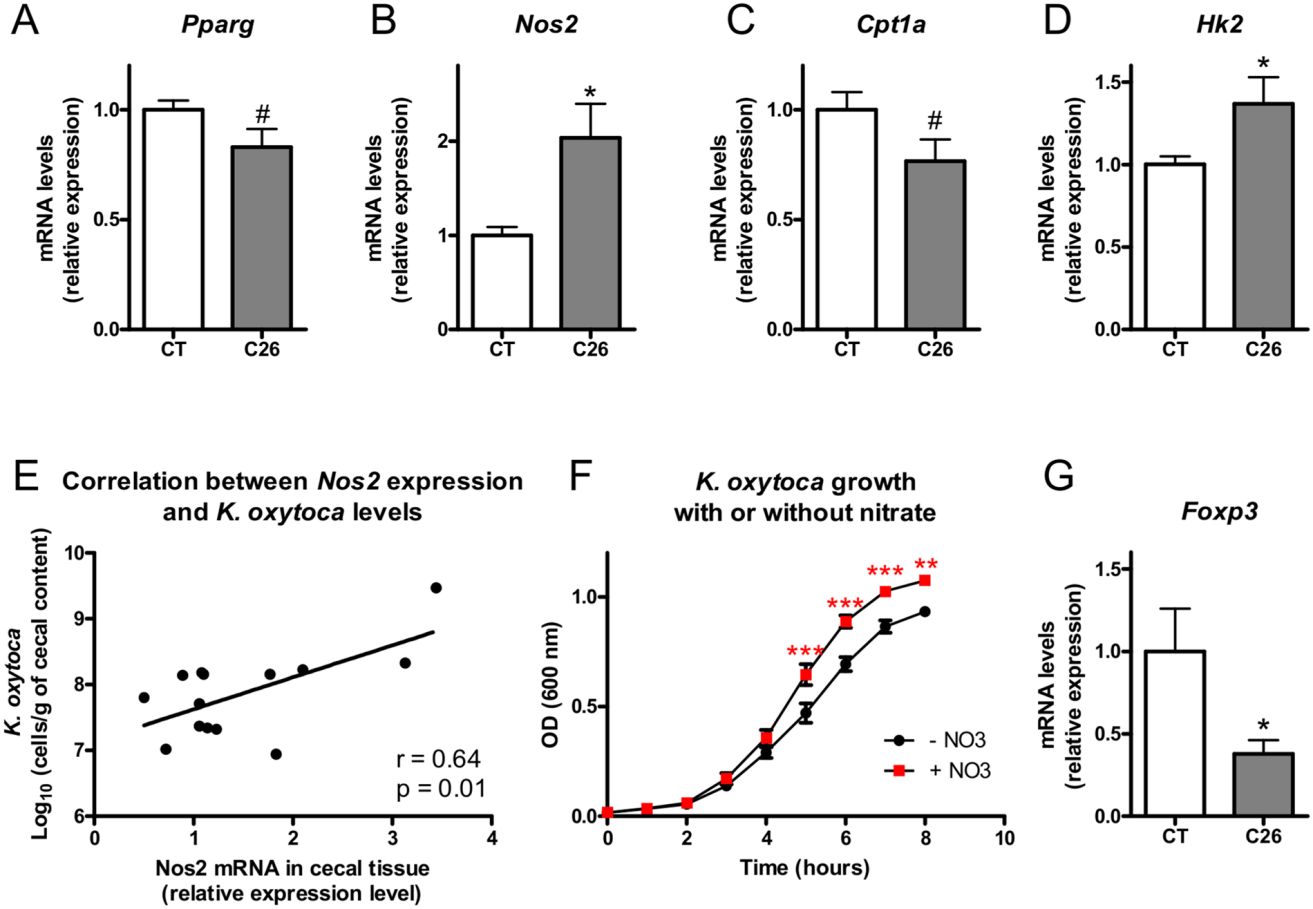
Mechanisms of emergence of *Klebsiella oxytoca* in cachectic mice. (**A**–**D**) Cecal *Pparg*, *Nos2*, *Cpt1a*, and *Hk2* expression in sham-injected mice (CT) and C26-injected mice (C26), n = 7–8. (**E**) Pearson correlation between cecal *K*. *oxytoca* levels and cecal *Nos2* expression, n = 14. (**F**) Optical density (OD) over time of a simplified medium enriched or not with nitrate and inoculated with *K*. *oxytoca*. This graph is the result of three independent experiments performed in duplicate. (**G**) Cecal *Foxp3* expression in sham-injected mice (CT) and C26-injected mice (C26), n = 7–8. Data are presented as mean ± SEM. ^#^p < 0.1; *p < 0.05; ***p < 0.001.

Interestingly, a bloom of *Enterobacteriaceae* at the expense of the *Ruminococcaceae* and *Lachnospiraceae* families has also been recently described by Byndloss and their colleagues after antibiotic treatment^22^. These authors demonstrated that a reduction in these butyrate-producing microbes by antibiotic treatment is driving a reduced PPAR-γ signaling, leading to reduced β-oxidation, glycolytic switch and increased expression of *Nos2* (encoding the inducible nitric oxide synthase, iNOS). Finally, they showed that this host-derived nitrate, as a respiratory electron acceptor, can then foster the growth of *Escherichia coli*^22,23^. In accordance with their findings, we found that in cachectic mice, *K*. *oxytoca* expansion was associated in the cecal tissue with a reduced PPAR-γ signaling (as suggested by a reduced *Pparg* expression and increased *iNOS* expression, a target gene of PPAR-γ^22,24^), reduced β–oxidation (as suggested by *Cpt1a*, encoding the carnitine palmitoyl-transferase 1a, which deficiency results in a decreased rate of β-oxydation^25^) and higher glycolysis (as suggested by *Hk2*, encoding the hexokinase 2) (Fig. 3A–D). Hk2 catalyzes the first step of glycolysis in the intestinal tissue and is upregulated at the transcriptional level during glycolytic switch^26,27^. We also found out that cecal *Nos2* expression was strongly correlated with cecal *K*. *oxytoca* levels (Fig. 3E). To further strengthen these findings, we asked the question whether nitrate could confer a growth advantage to *K*. *oxytoca*. Indeed, supplementation of a simplified growth medium with nitrate fostered the growth of *K*. *oxytoca* (Fig. 3F).

Byndloss and their colleagues further established that a reduction in the PPAR-γ signaling was necessary but not sufficient for increasing *Enterobacteriaceae* members. Indeed, a depletion in regulatory T cells (T_regs_) was needed as a second trigger for such an increase to occur. Concordantly, in cachectic mice, we found a reduction in the cecal T_regs_ pool (as measured by their surrogate marker, the transcription factor *Foxp3*) (Fig. 3G).

Of note, these alteration in PPAR-γ signaling and T_regs_ reduction could not be explained only by the reduced food intake as demonstrated by the pair-feeding experiment (Fig. S2). *Pparg* expression (as well as its target *Nos2*) was unsurprisingly decreased as caloric restriction was previously report to influence *Pparg* expression^28,29^. However, *Cpt1a*, *Hk2* and *Foxp3* expression were not affected.

Altogether, these findings strongly suggest that *K*. *oxytoca* expansion results from a cooperation between, on the one hand, the decreased abundance in the *Ruminococcaceae*, *Lachnospiraceae* and *Porphyromonadaceae* families accompanied by a reduced PPAR-γ signaling, and, on the other hand, the reduced cecal T_regs_ pool.

### *Klebsiella oxytoca* as a gut pathobiont

**Figure 4:**
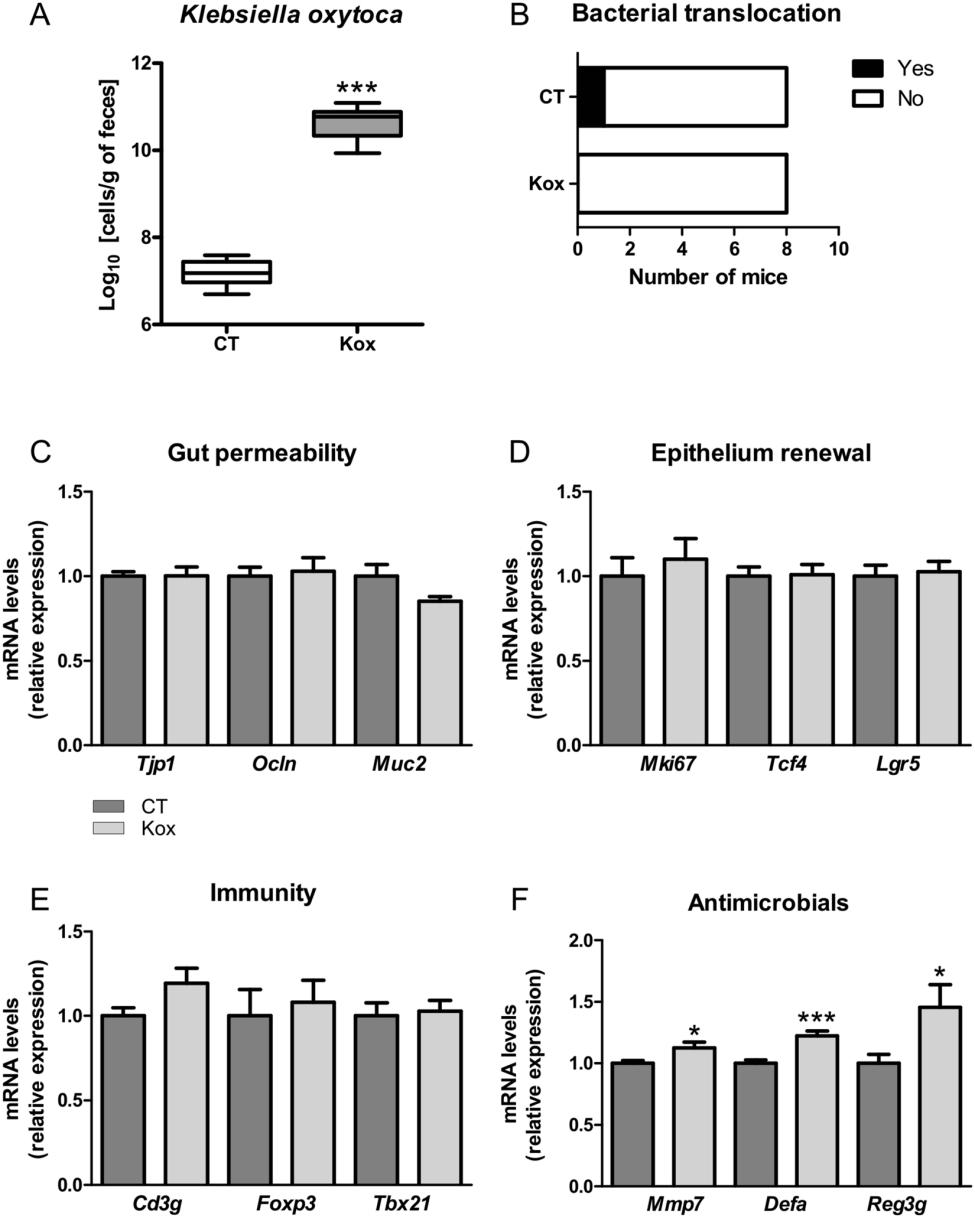
*Klebsiella oxytoca* does not affect gut barrier function in healthy mice. (**A**) Fecal *K*. *oxytoca* levels in healthy mice receiving the vehicle (CT) or *K*. *oxytoca* in their drinking bottle (Kox). (**B**) In black, number of mice for which coliform bacteria were detected in their mesenteric lymph nodes (MLN). (**C-F**) Ileal mRNA expression levels of key markers involved in gut permeability, epithelium renewal, mucosal immunity and antimicrobial peptide production. Data are presented as whiskers plots with minimal and maximal values (A) or mean ± SEM (**C–F**). N = 7–8. *p < 0.05; ***p < 0.001.

We next sought to determine the contribution of *K*. *oxytoca* to the intestinal alterations found in mice with cancer-related cachexia and, *in fine*, to cancer cachexia itself. Daily force-feeding can be a source of stress, especially for diseased mice. We therefore evaluated whether administration of *K*. *oxytoca* through the drinking water would be an appropriate method to increase *in vivo K*. *oxytoca* levels. *In vitro* experiments demonstrated that *K*. *oxytoca*’s viability is not affected by −80 °C storage (up to 3 weeks) (Fig. S3A) and that the levels of the bacterium remain stable when administered in the drinking water of mice for 24 hours (Fig. S3B). Administration of *K*. *oxytoca* to healthy mice for 9 days resulted in a 3.5-log increase in fecal levels of *K*. *oxytoca* (Fig. 4A). These observations validate the administration mode of the bacterium to mice.

In healthy mice, *K*. *oxytoca* did not induce a translocation of coliform bacteria to the mesenteric lymph nodes (MLN) (Fig. 4B). In accordance with this result, *K*. *oxytoca* did not affect the mRNA expression levels of several key markers involved in the gut permeability (namely, Zonula Occludens-1, Occludin and Mucin-2, Fig. 4C). It also did not affect the mRNA expression levels of several key markers involved in epithelium renewal (namely, Ki67, the T-cell specific transcription factor 4, TCF4, and the Leucin-rich repeat-containing G-protein coupled receptor 5, Lgr5) and those reflecting mucosal immunity (namely, CD3g, Foxp3 and Tbet) (Fig. 4D–E). A modest increase was found when examining the expression of antimicrobial peptides and associated markers (namely, the matrix metallopeptidase 7, the alpha-defensins and the regenerating islet derived protein 3 gamma, Reg3γ) (Fig. 4F). Altogether, these data allowed us to conclude that *K*. *oxytoca* does not affect gut barrier function in healthy mice.

**Figure 5:**
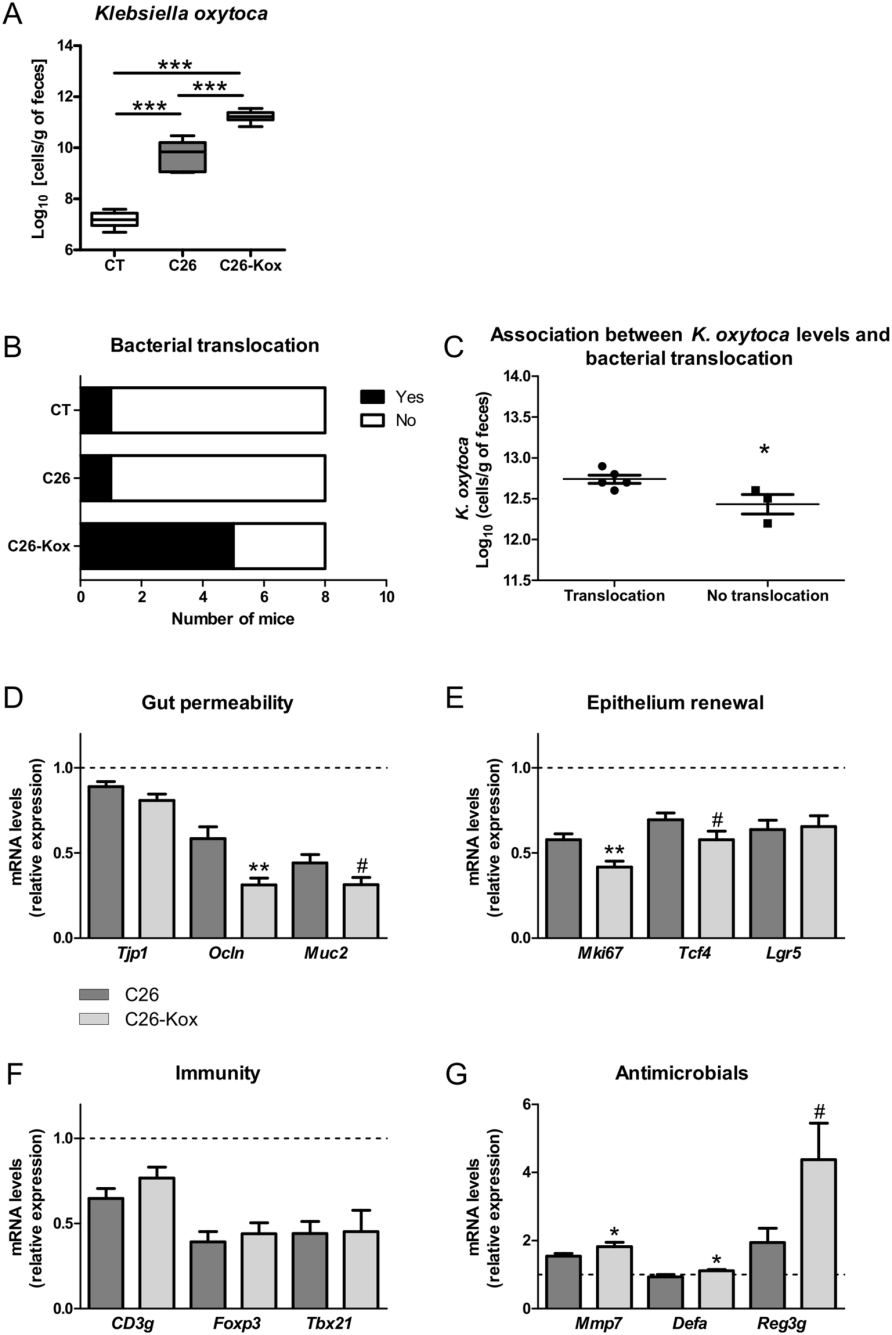
*Klebsiella oxytoca* hampers gut barrier function in cachectic mice. (**A**) Fecal *K*. *oxytoca* levels in healthy mice receiving the vehicle (CT), in C26-transplanted mice receiving the vehicle (C26) and in C26-transplanted mice receiving *K*. *oxytoca* in their drinking bottle (C26-Kox). (**B**) In black, number of mice for which coliform bacteria were detected in their mesenteric lymph nodes (MLN). (**C**) Fecal *K*. *oxytoca* levels in mice displaying coliform bacteria in their MLN and in mice free of coliform bacteria in their MLN, within the C26-Kox group. (**D**–**G**) Ileal mRNA expression levels of key markers involved in gut permeability, epithelium renewal, mucosal immunity and antimicrobial peptide production. Data are presented as whiskers plots with minimal and maximal values (A) or mean ± SEM (**C–G**). The dotted line indicates the expression level in sham-injected mice. N = 7–8. ^#^p < 0.1; *p < 0.05; **p < 0.01; ***p < 0.001.

In cachectic mice, we confirmed that *K*. *oxytoca* levels are increased. The administration of *K*. *oxytoca* in the drinking water induced an additional rise in its fecal levels (Fig. 5A). Analysis of the results of Figs 4A and 5A with an ANOVA model revealed a synergistic effect of exogenous administration of *K*. *oxytoca* and cachexia on fecal *K*. *oxytoca* levels (p-value interaction: 9.4 × 10^−8^). We therefore concluded that cachectic mice display an impaired resistance to colonization with an exogenous source of *K*. *oxytoca*.

When cachectic mice were administered *K*. *oxytoca*, we found an increased bacterial translocation in the MLN of these mice (χ² test, p = 0.04, Fig. 5B). *K*. *oxytoca* and *Enterobacter* sp. were among the bacteria that translocated to the MLN (Table S2). Interestingly, within the group of cachectic mice receiving *K*. *oxytoca*, mice displaying translocation of coliform bacteria showed a higher fecal content in the bacterium compared to mice free of coliform bacteria in the MLN (Fig. 5C). In accordance with these results, the expression of several key markers of the gut barrier function was affected by *K*. *oxytoca*, such as Occludin, Mucin-2, Ki67 and TCF4 (Fig. 5D–F). Similarly to what was observed in healthy mice, the expression of antimicrobial peptides and associated markers were increased by the administration of *K*. *oxytoca* (Fig. 5G).

As we found out that *K*. *oxytoca* can worsen the gut barrier dysfunction in cachectic mice, we wondered whether *K*. *oxytoca* could also impact cancer progression and other cachectic features. However, neither tumor growth nor any of the cachectic features evaluated in these mice (namely body weight change and muscle atrophy markers) were significantly impacted by *K*. *oxytoca* supplementation (Table 2).

**Table 2:** Body weight gain, tumor weight and expression of atrophy markers in the gastrocnemius of mice receiving a sham injection and receiving the vehicle in their drinking bottle (CT), mice transplanted with C26 cells and receiving the vehicle in their drinking bottle (C26) and mice transplanted with C26 cells and receiving *Klebsiella oxytoca* in their drinking bottle (C26-Kox).

Weights (g)	CT	C26	C26-Kox
Body weight gain	1.26 ± 0.36	−2.21 ± 0.28^***^	−2.21 ± 0.33^***^
Weight of the tumor	—	0.28 ± 0.02	0.25 ± 0.02
**mRNA levels**	**CT**	**C26**	**C26-Kox**
*Fbxo32*	1.00 ± 0.10	18.35 ± 2.20^***^	16.31 ± 2.59^***^
*Trim63*	1.00 ± 0.07	17.80 ± 2.56^***^	15.58 ± 2.82^***^
*Ctsl*	1.00 ± 0.04	3.76 ± 0.74^*^	3.36 ± 0.82^*^
*Map1lc3a*	1.00 ± 0.03	2.39 ± 0.17^***^	2.22 ± 0.26^***^

Data are presented as mean ± SEM. *p < 0.05; ***p < 0.001.

## Discussion

So far, most of the research concerning the role of the gut microbiota in the management of metabolic disorders has been performed in the context of overnutrition^30,31^. The gut microbiota might also play a role at the other end of the spectrum, in metabolic and immune alterations associated with undernutrition^20^. In this context, we initiated an original axis of research ten years ago focusing on the causal role and therapeutic potential of the gut microbiota in the metabolic alterations associated with cancer progression, usually defined as cancer cachexia. We found out that preclinical models of cancer cachexia were characterized by intestinal dysbiosis, with one of the main traits being an increase in *Enterobacteriaceae*^15,18^. Following up on this finding, we report here that the main *Enterobacteriaceae* species increased in cancer cachexia is *K*. *oxytoca* which behaves as a gut pathobiont. We also highlight in the present study by which mechanisms such a pathobiont can emerge in the context of cancer cachexia.

The *Enterobacteriaceae* family encompasses gram-negative facultative enteropathogens and pathobionts. *Enterobacteriaceae* reside in the gut at low levels and are localized in close proximity to the mucosal epithelium due to their relative higher tolerance to oxygen diffused from the epithelium^32^. Bloom of *Enterobacteriaceae* has been described in various pathological contexts, such as inflammatory bowel diseases, obesity, colorectal cancer, celiac disease and antibiotic treatment^32,33^. Several factors can contribute to the emergence of these bacteria, such as for instance, an increased nitrate production, higher levels of oxygen in the gut, release of phospholipids and sialic acid, depletion of microbiota-derived inhibitory products and increased nutrient availability^21,32^.

Host-derived nitrate can result from the reaction of superoxide radicals with nitric oxide, produced by the iNOS. Such nitrate can boost the growth of *E*. *coli* through its use as a terminal electron acceptor for the anaerobic respiration^23^. Beside this function, nitrate is a source of ammonium for biosynthesis and can generate proton-motive force for energy through its respiration^34^. Byndloss and colleagues highlighted last year a complex framework for the lower colonization resistance to *Enterobacteriaceae* after antibiotic treatment^22^. In brief, antibiotic treatment depletes butyrate-producing bacteria, thereby changing host cell metabolism (characterized among others by reduced PPAR-γ signaling and metabolic switch from β-oxidation to anaerobic glycolysis) to elevate iNOS synthesis and reduce host oxygen consumption. Thereby, antibiotics raise the concentration of host-derived respiratory electron acceptors such as nitrate, which can drive the expansion of *Enterobacteriaceae*^21,22^.

**Figure 6:**
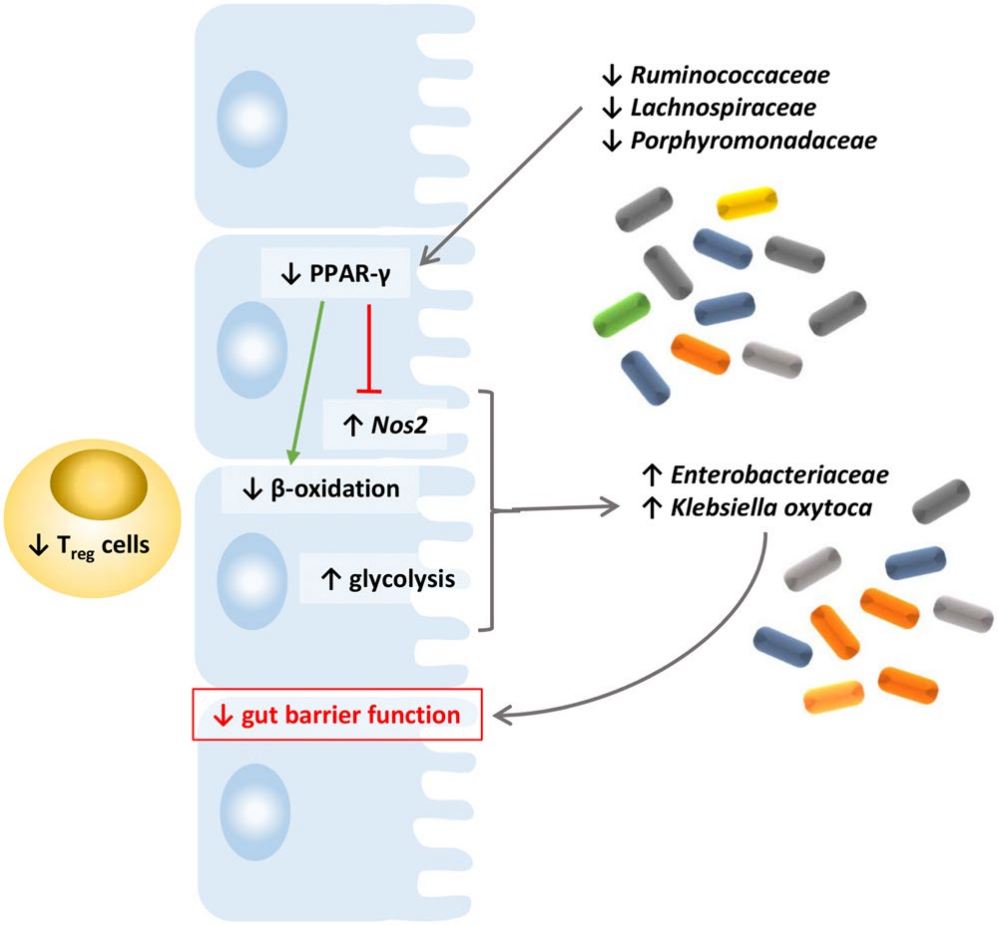
Based on our findings and previous literature in the field, we propose a conceptual framework for the emergence of *K*. *oxytoxa* in cachectic mice. In such framework, reduction in three butyrate-producing microbial families (*Ruminococcaceae*, *Lachnospiraceae* and *Porphyromonadaceae*) fosters a reduced PPAR-γ signaling, which leads to an increased *iNOS* expression. This reduced PPAR-γ signaling promotes a glycolytic switch (higher glycolysis and reduced β-oxidation), that together with a reduced T_regs_ abundance, allows the emergence of *K*. *oxytoca*. Host-derived nitrate production also contributes to the growth of the bacterium.

Building up on this conceptual framework for colonization resistance, we reasoned that the emergence of *Enterobacteriaceae* in cancer cachexia could be driven by similar factors (Fig. 6). In accordance with this hypothesis, we found out that three butyrate-producing families (*Ruminococcaceae*, *Lachnospiraceae* and *Porphyromonadaceae*^35^) are strongly and negatively correlated with the bloom of *Enterobacteriaceae*. These microbial changes are associated with alterations of gut epithelial metabolism and immunity, namely markers reflecting a reduction in PPAR-γ signaling, an increase in iNOS activity, a reduced β-oxidation and an increased glycolysis. Finally, we demonstrated that nitrate confers a growth advantage to *K*. *oxytoca*.

From our data, we concluded that although anorexia seems to play a role in the modification of the cecal PPAR-γ signaling, it does not drive alone the expansion of *K*. *oxytoca* found in cachectic mice. One explanation might involve the cecal T_regs_ pool. *Foxp3* expression, a marker of the T_regs_ pool, was decreased in cachectic mice but not affected in mice pair-fed to cachectic mice versus mice pair-fed to control mice. Byndloss and colleagues showed that a shrinkage in T_regs_ pool, concomitantly with a reduction in butyrate-producing members and PPAR-γ signaling, is needed to recapitulate the bloom of *Enterobacteriaceae*^22^. Therefore, the absence of effect of anorexia on the T_regs_ pool might explain the lack of expansion of *K*. *oxytoca* in pair-fed mice, a hypothesis that would need to be experimentally confirmed.

The term ‘pathobionts’ has been suggested to describe resident microbes with pathogenic potential. Pathobionts are innocuous to the host under normal conditions, distinct from traditional pathogens which may cause disease even in healthy hosts^36^. According to this definition, our data clearly show that *K*. *oxytoca* behaves as a gut pathobiont in cancer cachexia. Indeed, *K*. *oxytoca* worsened the gut barrier dysfunction in cachectic mice whereas no effects on the gut barrier function were observed when administered to healthy mice. To our knowledge, so far, *K*. *oxytoca* has been described as a gut pathobiont in only one other pathology, namely antibiotic-associated hemorrhagic colitis^37^. In this context, a toxin produced by *K*. *oxytoca* was isolated and identified as tilivalline. The authors showed that tilivalline induced apoptosis in cultured human cells *in vitro* and disrupted epithelial barrier function *in vivo*^38^. However, the *K*. *oxytoca* strain administered to the mice did not present any sign of cytotoxin production such as tilivalline in the tested conditions (Fig. S4) suggesting that, in cancer cachexia, the mechanism explaining its pathobiont character is independent from tilivalline. Future research will need to determine the nature of the molecular mediator(s) driving the pathobiont ability of this *K*. *oxytoca* strain.

Strikingly, *K*. *oxytoca* administration hampers gut barrier function in cachectic mice but did not worsen any of the other cachectic features that we assessed. From this observation, it is tempting to speculate that the bloom of *Enterobacteriaceae* does not contribute significantly to the systemic alterations found in cancer cachexia. However, we showed that supplementation with a synbiotic approach reduced the expansion of *Enterobacteriaceae*, reduced cancer cell proliferation in the liver, partially protected cachectic mice from muscle atrophy and increased survival^15^. One explanation could be that a combination of microbial changes (namely a full reversal of the intestinal dysbiosis) is needed to observe a benefit for synbiotics in cachexia. In addition, we can also not exclude that exogenous administration of *K*. *oxytoca* does not fully mimic the endogenous *K*. *oxytoca* expansion which is associated with a defect in the intestinal immune response such as for example the reduced T_reg_ cell pool.

The translational potential of our findings extends beyond cancer cachexia itself. In clinical practice, cancer cachexia is often worsened by anticancer therapy^8^, which exerts drastic antimicrobial effects and can drive pathogen overgrowth^39^. Antibiotics are often administrated during anticancer therapy to prevent or treat life-threatening infections^39^. Antibiotics are also part of the conditioning regimen for hematopoietic stem cell transplantation (often used as a treatment for hematological malignancies), where the gut microbiota appears to play a role, especially in the development of the main complications, the graft-versus-host disease and infections^40^. In a study involving 80 recipients of allo-hematopoietic stem cell transplantation, the post-engraftment microbiota of subjects who died differed significantly from the microbiota of patients who survived, harboring a greater abundance of *Enterobacteriaceae* and a lower abundance of *Lachnospiraceae*^41^. Our data suggested that colonization resistance to *Enterobacteriaceae* (and Proteobacteria in general) is decreased by the presence of the cancer alone. In clinical practice, such mechanism could therefore act conjointly with the antibiotics and anticancer treatments to foster the emergence of life-threatening pathogens.

Altogether, our findings established a role for *K*. *oxytoca* as a gut pathobiont in cancer cachexia and exposed new mechanisms of emergence of this bacterium in cancer cachexia, thereby further expanding the conceptual framework underlying the concept of colonization resistance to *Enterobacteriaceae*. We believe this set of studies constitutes a strong progression in the field of gut microbiota in cancer cachexia, by dissecting the mechanism of emergence of one bacterium and investigating its role in this severe disease. Clinical studies investigating the gut microbiota composition in cancer patients with and without cachexia are currently ongoing and should reveal key information about the translational potential of our findings.

## Material and Methods

### Cell culture

Colon carcinoma 26 (C26) cells were maintained in DMEM high glucose medium supplemented with 10% fetal bovine serum (PAA clone, PAA, Austria), 100 µg/ml streptomycin and 100 IU/ml penicillin (Gibco, Belgium) at 37 °C with 5% CO_2_.

### Mouse experiments

Male CD2F1 mice (7 weeks old, Charles River Laboratories, Italy) were kept in specific pathogen free conditions and housed in individually ventilated cages with a 12 h light/dark cycle and fed an irradiated chow diet (AO4–10, 2.9 kcal/g, Safe, France). After one week acclimatization, either a saline solution or C26 cells (1 × 10^6^ cells in 0.1 ml saline) were injected subcutaneously. All C26-injected mice displayed a tumor mass observable at day 7. Food intake and body weight were recorded. Eight mice were randomly assigned in each group based on their body weight on the day of cell injection.

The pair-feeding experiment was composed of 4 groups of mice: CT group (sham-injected and fed *ad libitum*), C26 group (receiving an injection of C26 cancer cells and fed *ad libitum*), PFtoCT group (sham-injected and fed the mean amount consumed by the CT mice) and PFtoC26 group (sham-injected and fed the mean amount consumed by the C26 mice). Pair-fed mice received daily in 2 equal portions the amount of food consumed by the group they were matched to, with one week delay.

When appropriate, mice received *K*. *oxytoca* or the vehicle in their drinking bottles at a concentration of 10^9^ CFU/ml, from day 1 after cell injection until the end of the experiment. Solutions were replaced every evening.

Nine to ten days after cancer cell injection, fresh feces were collected, mice were fasted from 7AM to 1PM (except for one experiment) and tissue samples and cecal content were harvested following anesthesia (isoflurane gas, Abbot, Belgium). Tissues were weighed and frozen in liquid nitrogen. All samples were stored at −80 °C until further analyses.

### Ethical statement related to mouse experiments

The experiments were approved by and performed in accordance with the guidelines of the local ethics committee from the Université catholique de Louvain. Housing conditions were as specified by the Belgian Law of 29 May 2013, regarding the protection of laboratory animals (agreement no LA1230314).

### Microbial experiments

#### Isolation and identification of coliform bacteria from cachectic mice

Feces from four cachectic mice were plated on MacConkey agar medium (BD Difco, USA) and plates were incubated at 37 °C. Isolates (15–16 per mouse) were cultured in tryptic soy broth (Sigma-Aldrich, USA). Stocks of pelleted bacteria suspended in culture medium with 15% glycerol were stored at −80 °C. All isolates were identified by full length 16S rRNA gene sequencing, a method that has been validated for identification of *Klebsiella*^42^. Briefly, DNA of these bacteria was extracted using the QI Amp Fast DNA stool Minikit (Qiagen, Germany) and amplified using 8 F and 1391 R primers (primer sequences in Table S2). The PCR products were purified and subjected to sequencing using the services of DNAVision (Belgium) or Eurofins Genomics (Germany). The sequences were compared with those available in the RDP and EZ BioCloud databases.

#### Growth of K. oxytoca in a medium with or without nitrate

In anaerobic conditions, 100 ml of a No-Carbon E medium (NCE) supplemented with trace elements^23^ and with or without sodium nitrate (40 mM) was inoculated with 1 ml of saturated culture of *K*. *oxytoca* and placed at 37 °C. 1 ml of this solution was sampled at each time point to measure the OD at 600 nm.

#### Growth of K. oxytoca for *in vivo* administration

*K*. *oxytoca* was grown in tryptic soy broth. Bacterial culture was centrifuged, supernatant removed and pelleted bacteria stored at −80 °C. Bacterial numeration was performed after thawing by plating on tryptic soy agar (Sigma-Aldrich, USA), and the bacteria were suspended in the drinking water (vehicle) at an adequate concentration. Identity of the bacterium was checked for each batch using PCR with primers targeting *K*. *oxytoca* and visual inspection of the PCR product on agarose gel (Fig. S5). Picture was acquired with a common digital camera. No further processing were applied, beyond a croping of the borders of the image.

#### Isolation of coliform bacteria from the mesenteric lymph nodes (MLN)

Mesenteric lymph nodes were harvested and homogenized in 1 ml sterile PBS using a dounce. 200 µl of the solution were plated on MacConkey agar plates and incubated in aerobic and anaerobic conditions at 37 °C. DNA was directly amplified from an aqueous bacterial suspension using 8 F and 1391 R primers (primer sequences in Table S2). The PCR products were purified and subjected to sequencing using the services of Eurofins Genomics (Germany). The sequences were compared with those available in the RDP and EZ BioCloud databases.

#### Evaluation of cytotoxin production

Evaluation of cytotoxin production was performed following the principle described by Darby and colleagues^43^. Briefly, HeLa cells (10 000 cells/well, 96-well plate) were incubated in triplicate in presence of various dilutions of bacterial supernatant. After 48 h, cell viability was assessed using a MTT test and data were expressed as percentage of the signal detected for cells incubated with the same dilution of the bacterial medium. The experiment was repeated with three independent bacterial isolates. The MTT assay is based on the principle that metabolically active cells will cleave yellow thiazolyl blue tetrazolium bromide (MTT, 0.5 mg/ml) to form purple formazan crystals. The formazan absorbance was measured at 570 nm, from which a background value, measured at 650 nm, was subtracted.

### Tissue mRNA analyses

The isolation of RNA, preparation of complementary cDNA and real-time polymerase chain reaction were performed as previously described^15^ (primer sequences in Table S2).

### Gut microbiota analyses

Genomic DNA was extracted from the cecal content or feces using the QIAamp DNA Stool Mini Kit, including a bead-beating step. Absolute quantification of the *K*. *oxytoca* species levels were performed using qPCR (primer sequences in Table S2). The samples were PCR-enriched for the V5-V6 region of the 16S rRNA gene and then underwent a library tailing PCR (primer sequences in Table S2). The amplicons were purified, quantified and sequenced using an Illumina MiSeq to produce 2 × 300 bp sequencing products. Initial quality-filtering of the reads was conducted with the Illumina Software, yielding an average of 164 603 pass-filter reads per sample. Quality scores were visualized and reads were trimmed to 220 bp (R1) and 200 bp (R2). The reads were merged with the merge-Illumina-pairs application^44^. For all samples, a subset of 25 000 reads was randomly selected using Mothur 1.32.1^45^. The UPARSE pipeline implemented in USEARCH v10.0.240^46^ was used to further process the sequences. Putative chimaeras were removed using the *cluster_otus* command. Non-chimeric sequences were subjected to taxonomic classification using the RDP MultiClassifier 1.1 from the Ribosomal Database Project^47^. The phylotypes were computed as percent proportions based on the total number of sequences in each sample. Significantly affected phyla and families were identified by Welch’s t-test. The p-value was adjusted to control for the false discovery rate (FDR) for multiple tests according to the Benjamini and Hochberg procedure^48^. The 16S rDNA sequencing dataset generated and analyzed for this study can be found in the SRA database (project ID: SRP144622, https://www.ncbi.nlm.nih.gov/sra/SRP144622).

### Statistical analyses

Outliers were removed using the Grub’s test. The statistical significance of differences between groups was assessed using Student’s t-test when comparing two groups or one-way ANOVA followed by Tukey’s multiple comparison tests when comparing several groups. Two-way ANOVA followed by Bonferroni post-tests was used to assess the significance of two independent variables for one dependent variable. Multiple correlation analyses based on Spearman correlation were done in R, with adjustment of the p-value according to the Benjamini and Hochberg procedure. The heatmap was drawn using the *heatmap*.*2* function in the gplots package (v2.17.0) as well as the RColorBrewer package (v1.1-2). Co-abundance network was built using CoNet and Cytoscape (v3.6.1)^49,50^. Statistical analyses were performed using GraphPad Prism 5.0 and R^51^. P < 0.05 was considered statistically significant.

## Electronic supplementary material

Supplementary information

## Data Availability

The 16S rDNA sequencing dataset generated and analyzed for this study can be found in the SRA database (project ID: SRP144622, https://www.ncbi.nlm.nih.gov/sra/SRP144622).
