# Supplementary material for: Klebsiella oxytoca expands in cancer cachexia and acts as a gut pathobiont contributing to intestinal dysfunction
Source: Sci Rep. 2018 Aug 17;8:12321. doi: 10.1038/s41598-018-30569-5 (PMC6098145; doi:10.1038/s41598-018-30569-5)
Supplement: Supplementary file 1 — Supplementary information [file 41598_2018_30569_MOESM1_ESM.pdf]

Klebsiella oxytoca expands in cancer cachexia and acts as a gut pathobiont contributing to intestinal dysfunction

Sarah A. Pötgens[1]†, Hélène Brossel[1]†, Martina Sboarina[1], Emilie Catry[1], Patrice D. Cani[1,2], Audrey M. Neyrinck[1], Nathalie M. Delzenne[1×], Laure B. Bindels[1×#]

[1]Metabolism and Nutrition Research Group, Louvain Drug Research Institute, Université catholique de Louvain, Brussels, Belgium.

[2]Walloon Excellence in Life Sciences and BIOtechnology (WELBIO), Louvain Drug Research Institute, Université catholique de Louvain, Brussels, Belgium

# Correspondence: Laure B. Bindels

laure.bindels@uclouvain.be

†× These authors contributed equally to this work.

ORCID: NMD 0000-0003-2115-6082, LBB 0000-0003-3747-3234.

Running title: *Klebsiella oxytoca* in cancer cachexia

Keywords: cancer cachexia, Proteobacteria, *Enterobacteriaceae*, *Klebsiella oxytoca*, gut barrier, gut microbiota, colonization resistance, PPAR-γ.

Figure S1. Co-abundance network illustrating the interactions among bacterial families in the control and cachectic mice (n=13).

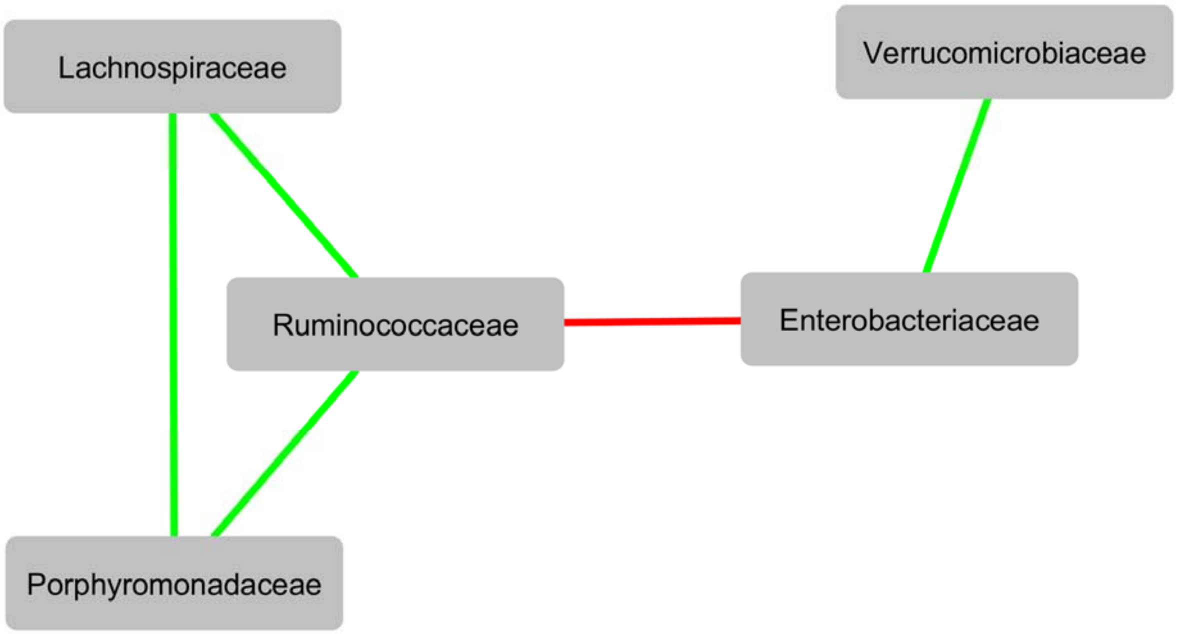

The co-abundance network was built using the CoNet App in Cytoscape following author's tutorial (http://psbweb05.psb.ugent.be/conet/microbialnetworks/conet_new.php). The line connecting two nodes represents the correlation between the connected nodes: green represents a positive correlation, and red represents a negative correlation. Only lines corresponding to correlations with corrected p-value below 0.05 were drawn.

Main CoNet parameters

- taxa below a minimum occurrence of 5 across samples were lumped into a garbage taxon.
- 30 top edges
- 100 iterations (methods: Pearson, Spearman, Mutual Information, Bray Curtis and Kullback-Leibler dissimilarity)
- Bootstrapping and p-value merging using the Brown's method
- Benjamini-Hochberg multiple test correction

Figure S2. Cecal *Pparg*, *Nos2, Cpt1a, Hk2* and *Foxp3* expression in sham-injected mice pair-fed to control mice (PFtoCT) and pair-fed to C26-injected mice (PFtoC26), n = 7-8. Data are presented as mean ± SEM. ** $p < 0.01$.

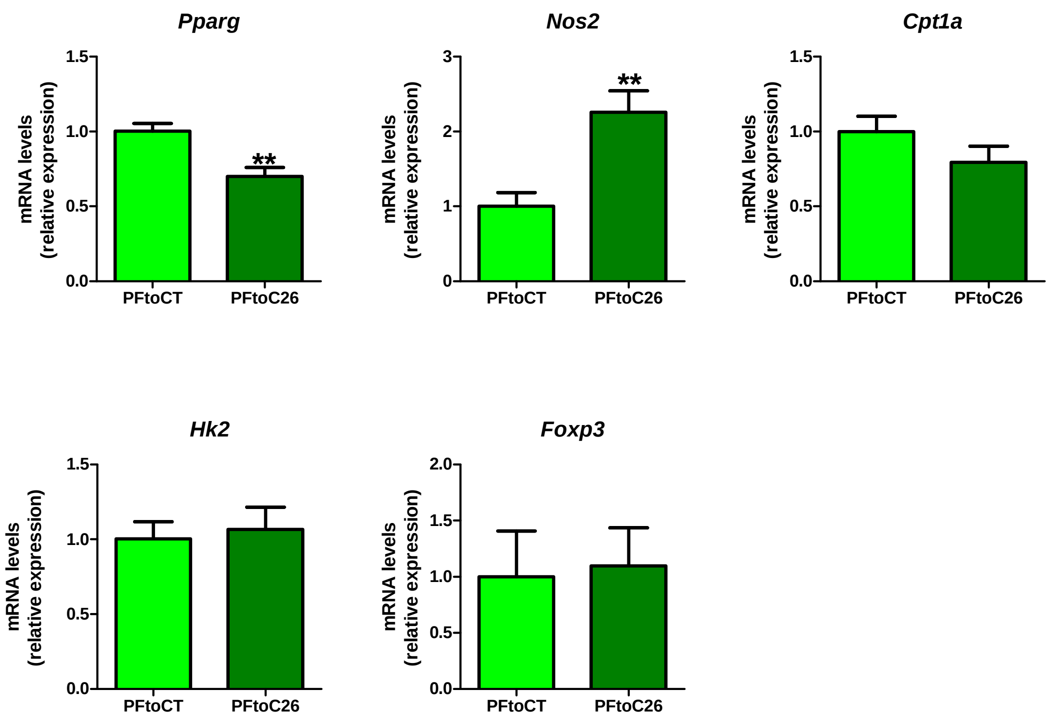

Figure S3. *In vitro* tests to validate the administration mode of *Klebsiella oxytoca* to mice. **(A)** Concentration of live bacteria, in colony-forming units (CFU) per ml of vehicle, of fresh aliquots and after one-week freezing and three-week freezing at -80°C. **(B)** Concentration of live bacteria, in colony-forming units (CFU) per ml of vehicle, over time in a mouse drinking bottle. These graphs are the result of three independent experiments performed in duplicate. Data are presented as mean ± SEM.

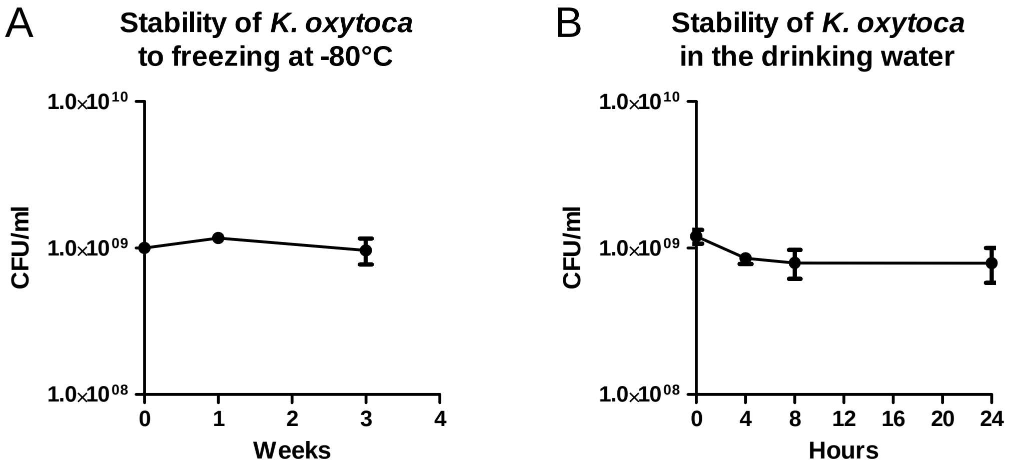

Figure S4. Evaluation of cytotoxin production by the *K. oxytoca* isolate used in the *in vivo* experiment. Viability of HeLa cells incubated in presence of various dilutions of bacterial supernatant was measured by a MTT test and data were expressed as percentage of the signal detected for cells incubated with the same dilution of the bacterial medium, n = 3. Data are presented as mean ± SEM.

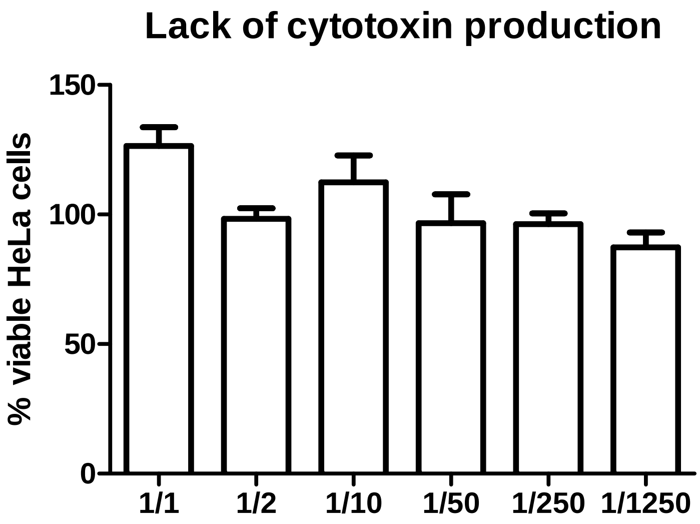

Figure S5. Identity of the bacterium was checked for each batch prepared for *in vivo* administration using PCR with primers targeting *K. oxytoca* and visual inspection of the PCR product on agarose gel. The expected size is 334 bp. K, standard *K. oxytoca* used as inoculate and whose 16S rRNA gene has been sequenced by Sanger sequencing; L1-L6, 6 different lots of bacteria; Bl, blank.

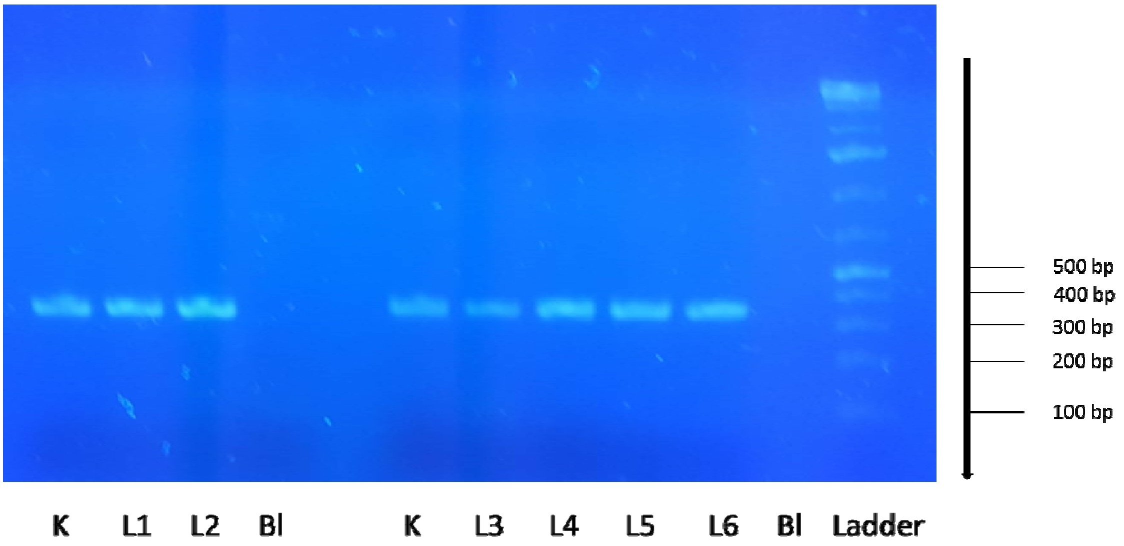

Picture issued from one gel, acquired with a common digital camera. No further processing were applied, beyond a croping of the borders of the image.

Table S1. Relative abundance of bacterial phyla and families in control (CT) and cachectic mice (C26).

Taxonomic name	rank	mean abundance (%)		SEM		P-value	Q-value
		CT	C26	CT	C26		
Firmicutes	phylum	56.304	19.747	1.409	2.394	0.000	0.000
Bacteroidetes	phylum	33.892	28.650	1.209	1.411	0.017	0.061
Verrucomicrobia	phylum	6.504	26.626	0.576	1.778	0.000	0.000
Actinobacteria	phylum	0.059	0.045	0.006	0.019	0.480	0.627
Deferribacteres	phylum	1.473	3.152	0.244	0.618	0.032	0.100
Proteobacteria	phylum	0.032	21.593	0.011	4.037	0.001	0.006
Cyanobacteria/Chloroplast	phylum	0.002	0.002	0.001	0.002	0.900	0.900
Lachnospiraceae	family	30.111	12.748	1.232	1.665	0.000	0.000
Ruminococcaceae	family	11.838	3.959	0.428	0.673	0.000	0.000
Clostridiaceae 1	family	0.019	0.129	0.011	0.104	0.329	0.506
Peptostreptococcaceae	family	0.000	0.001	0.000	0.001	0.351	0.506
Lactobacillaceae	family	0.618	0.247	0.147	0.065	0.064	0.186
Enterococcaceae	family	0.022	0.031	0.017	0.008	0.655	0.782
Staphylococcaceae	family	0.000	0.126	0.000	0.096	0.232	0.506
Erysipelotrichaceae	family	0.075	0.132	0.019	0.056	0.354	0.506
Porphyromonadaceae	family	33.881	28.647	1.206	1.412	0.017	0.061
Bacteroidaceae	family	0.009	0.002	0.005	0.001	0.264	0.506
Verrucomicrobiaceae	family	6.504	26.626	0.576	1.778	0.000	0.000
Coriobacteriaceae	family	0.056	0.044	0.006	0.018	0.545	0.672
Micrococcaceae	family	0.002	0.000	0.002	0.000	0.374	0.506
Microbacteriaceae	family	0.000	0.001	0.000	0.001	0.170	0.449
Bifidobacteriaceae	family	0.002	0.000	0.001	0.000	0.178	0.449
Deferribacteraceae	family	1.473	3.152	0.244	0.618	0.032	0.100
Enterobacteriaceae	family	0.029	21.589	0.010	4.037	0.001	0.006
Comamonadaceae	family	0.001	0.001	0.001	0.001	0.752	0.850
Burkholderiaceae	family	0.002	0.002	0.001	0.001	0.822	0.877
Sutterellaceae	family	0.000	0.001	0.000	0.001	0.351	0.506
Chloroplast	family	0.002	0.002	0.001	0.002	0.900	0.900

Table S2. Identification of bacterial isolates recovered from mesenteric lymph nodes of control (CT), C26 transplanted mice (C26) and C26 transplanted mice receiving K. oxytoca in their drinking water (C26-KO) using a selective medium for coliform bacteria.

**Identification/ Prevalence**	**CT**	**C26**	**C26-KO**
Klebsiella oxytoca	0/1	2/5	4/8
Enterobacter sp.	1/1	3/5	4/8

Table S3. Primers used in the study.

Target name	Alias/full name	Forward	Reverse
RPL19 (housekeeping gene)		GAAGGTCAAAGGGAATGTGTTCA	CCTTGTCTGCCTTCAGCTTGT
Fbxo32	atrogin 1, MAFbx, F-box protein 32	ATGCACACTGGTGCAGAGAG	TGTAAGCACACAGGCAGGTC
Ctsl	cathepsin L	GTGGACTGTTCTCACGCTCAAG	TCCGTCCTTCGCTTCATAGG
Cd3g	CD3g	TCTCTACTGGGCTCTCTCCAA	CCATCTCCAAGGAAACCAAC
Defa	alpha-defensins	GGTGATCATGAGACCCCAGCATCAGT	AAGAGACTAAAACTGAGGAGCAGC
Foxp3	forkhead box P3	TCCTTCCCAGAGTTCTTCCA	CGAACATGCGAGTAAACCAA
Hk2	hexokinase 2	GACCAGAGCATCCTCCTCAA	CCACATCCAGGTCAAACTCC
Mki67	Ki67	CAGACTTGCTCTGGCCTACC	GGTTGGCGTTTCTCCTCTTT
Map1lc3a	LC3	CACTGCTCTGTCTTGTGTAGGTTG	TCGTTGTGCCTTTATTAGTGCATC
Lgr5	Gpr49	TTGGAGAAAGGAGAGCTGGA	AGTGGGACGATCACGAGAAG
Mmp7	Matrix metallopeptidase 7	TGTTCCCGGTACTGTGATGT	CACAGCGTGTTCCTCTTTCC
Muc2	Mucin 2	ATGCCCACCTCCTCAAAGAC	GTAGTTTCCGTTGGAACAGTGAA
Nos2	inducible NO synthase	AAACTGTGTGCCTGGAGGTT	TCTCTGCCTATCCGTCTCGT
Trim63	MuRF1	ACGAGAAGAAGAGCGAGC	CTTGGCACTTGAGAGGAA
Ocln	occludin	ATGTCCGGCCGATGCTCTC	TTTGGCTGCTCTTGGGTCTGTAT
Reg3g	regenerating islet derived protein 3 gamma	TTCCTGTCCTCCATGATCAAA	CATCCACCTCTGTTGGGTTC
Tcf4	T-cell specific transcription factor 4	ATGGCAAACAGAGGAACTGG	GCCTGCTGAGAGTGAAGGAG
Tbx21	Tbet	CAGTGTGGAAAGGCAGAAGG	GGGCTGGTACTTGTGGAGAG
Tjp1	ZO1	TTTTTGACAGGGGGAGTGG	TGCTGCAGAGGTCAAAGTTCAAG
*Klebsiella oxytoca**		GATACGGAGTATGCCTTTACGGTG	TAGCCTTTATCAAGCGGATACTGG

*as reported by Kovtunovych et al, Res Microbiol 2003, annealing temperature of 59°C.

**Primers for 16S sequencing by Illumina MiSeq**	
V5F_Nextera	TCGTCGGCAGCGTCAGATGTGTATAAGAGACAG**RGGATTAGATACCC**
V6R_Nextera	GTCTCGTGGGCTCGGAGATGTGTATAAGAGACAG**CGACRRCCATGCANCACCT**
Forward indexing primer	*AATGATACGGCGACCACCGA*GATCTACAC[i5]TCGTCGGCAGCGTC
Reverse indexing primer	*CAAGCAGAAGACGGCATACGA*GAT[i7]GTCTCGTGGGCTCGG

16S-specific portion is indicated in bold, and the p5 and p7 flow cell adapters are shown in italics.

**Primers for 16S sequencing by Sanger**	
8F	AGAGTTTGATCCTGGCTCAG
1391R	GACGGGCGGTGWGTRCA
